# Simultaneous Imaging of Bio- and Non-Conductive Targets by Combining Frequency and Time Difference Imaging Methods in Electrical Impedance Tomography

**DOI:** 10.3390/bios11060176

**Published:** 2021-05-31

**Authors:** Xue Bai, Dun Liu, Jinzhao Wei, Xu Bai, Shijie Sun, Wenbin Tian

**Affiliations:** 1 School of Instrumentation and Optoelectronic Engineering, Beihang University, Beijing 100191, China; bxbuaabj@buaa.edu.cn (X.B.); weijinzhao@buaa.edu.cn (J.W.); xubai11@163.com (X.B.); sunsj@buaa.edu.cn (S.S.); 2 Beijing Advanced Innovation Center for Big Data-Based Precision Medicine, Beihang University, Beijing 100191, China; 3 College of Engineering, China Agricultural University, Beijing 100083, China; wenbin.tian@hotmail.com

**Keywords:** electrical impedance tomography, frequency difference, time difference, lung imaging

## Abstract

As a promising medical imaging modality, electrical impedance tomography (EIT) can image the electrical properties within a region of interest using electrical measurements applied at electrodes on the region boundary. This paper proposes to combine frequency and time difference imaging methods in EIT to simultaneously image bio- and non-conductive targets, where the image fusion is accomplished by applying a wavelet-based technique. To enable image fusion, both time and frequency difference imaging methods are investigated regarding the reconstruction of bio- or non-conductive inclusions in the target region at varied excitation frequencies, indicating that none of those two methods can tackle with the scenarios where both bio- and non-conductive inclusions exist. This dilemma can be resolved by fusing the time difference (td) and appropriate frequency difference (fd) EIT images since they are complementary to each other. Through simulation and in vitro experiment, it is demonstrated that the proposed fusion method can reasonably reconstruct both the bio- and non-conductive inclusions within the lung models established to simulate the ventilation process, which is expected to be beneficial for the diagnosis of lung-tissue related diseases by EIT.

## 1. Introduction

Electrical impedance tomography (EIT) is a non-invasive imaging technique in which the absolute value or the change of conductivity distributions are reconstructed [1,2,3,4,5,6]. The underlying rotational is based on generating a current density distribution inside the medium and measuring the resultant electromagnetic fields by means of sensors positioned circumferentially on the boundary [2]. Current excitation and voltage acquisition are repeated continuously and rapidly until all the independent electrode combinations are deployed. Attributed to its notable merits of high temporal resolution, non-radiation, low cost, and non-intrusive measurement, EIT has also gained a great deal of interest in monitoring rapid changes in multiphase flow and medical imaging [2,3,4,5,6].

Ever since medical interest in EIT increased in 1980s, EIT has been developed to be a promising medical imaging technique to attain useful diagnostic information where there are obvious conductive changes in the biological tissues and organs with changes in physiological conditions, such as breathing, blood flow, digestive, canceration, and nervous activity [7,8]. Up to now, the medical applications of EIT research have mainly focused on thoracic imaging. Among the organs contained in the thorax, large volumes of air are Amoved into and out of the lungs, leading to obvious conductivity changes during ventilation. Therefore, it is agreed that monitoring of breathing by imaging the lung is the most important physiological application of this technique. Monitoring ventilation by EIT has already been applied for managing mechanically ventilated patients as it is can guide the set-up of mechanical ventilator pressure, volume, and respiratory rate [9].

Most clinical and physiological research into the lung with EIT is conducted using the difference imaging method to obtain air distribution images of ventilation and perfusion in the chest. Static imaging, i.e., reconstructing the absolute conductivity distribution of interest from a single set of measurement, has already been abandoned because of the poor image quality. Time difference EIT (td-EIT) was the consensus on the performance figures of merits for EIT image reconstruction after it was proposed by Barber and Brown [10]. Meanwhile, it was tested successfully for lung imaging as it can ameliorate modeling errors contained in the measurement data [11]. By taking the merits of frequency dependence of biological tissue, frequency difference EIT (fd-EIT) aroused significant interest by injecting currents with two or more different frequencies [11,12,13,14,15,16,17,18]. Compared with the td-EIT imaging method, fd-EIT does not need the reference measurement from the past, so it tackles the issue in terms of the unavailable voltage data with respect to the reference measurement in a real clinic environment [11,12,13]. Subsequently, developing a multi-frequency EIT (mf-EIT) system is an important transition for the research of EIT. Remarkable work includes the Sheffield system [14], the UCL systems [15], the KHU systems [16,17], and the Edinburgh system [3].

Many lung pathologies, such as atelectasis (lung collapse), pneumothorax, pleural effusion, and pulmonary edema, explicitly indicate an abnormity of air or liquid content in the lung. For example, the “pleural effusion” model in EIT contains a high conductivity region due to the accumulation of liquid in the pleural cavity [9,18]. Generally, diagnosis of several lung pathologies, such as avoiding acute respiratory distress syndrome (ARDS) with assessment of extravascular lung water, which is also known as quantify pulmonary edema and which can be considered as a biomaterial, may benefit from the use of the fd-EIT imaging method [19]. However, fd-EIT imaging faces a main challenge. Biomaterials such as lung tissues are only shown in the reconstructed images as their conductivity varies with frequency of the injected current. Thus, for lung imaging, the reconstructed images of fd-EIT are not well characterized in terms of attaining explicit interpretation of the pulmonary monitoring and air content. For the td-image reconstruction, it can be expected that the biological materials would induce much smaller conductivity variation than that of the non-conductive materials, and thus the biomaterial distribution would be hardly seen in the reconstructed images due to the existence of the large amount of air. Another limitation is that most EIT signals from ventilation may overwhelm those from perfusion. Therefore, EIT on lung monitoring by using only fd-EIT or td-EIT cannot stratify certain clinical requirements. In medical imaging applications, the images of different modalities have already been fused to obtain reliable and accurate diagnoses [20]. Inspired by the image fusion concept, it is meaningful to investigate the possibility of improving the image quality by fusing the reconstructed images of the td-EIT and fd-EIT since it is convenient to acquire both the td-EIT and fd-EIT data simultaneously.

In this paper, to simultaneously image bio- and non-conductive targets by EIT, it is proposed to fuse the reconstructed images by fd- and td-EIT to reveal both the bio- and non-conductive materials distributions, where the wavelet-based fusion method is employed. At the beginning, the td- and fd-EIT imaging methods to image bio- or non-conductive material distributions are investigated. This provides an in-depth look at the limitations of td- and fd-EIT imaging. The wavelet transform was performed to decompose and fuse the reconstructed td- and fd-EIT images, with the performances of typical wavelet basis functions compared to attain optimized image quality. Since the inherent properties of the lungs during breathing are well matched to the application of the proposed method, the lung ventilation imaging was studied. For the lung imaging, a simulation study was carried out by considering a homogenous phantom of the human thorax with lungs simulated as real-shaped abnormal inclusions containing air. The proposed method was evaluated by imaging the simulated ventilation process of the lung. In the end, experiments were conducted to validate the proposed method by adopting an in vitro lung model made with a white gourd.

This paper is structured as follows: In Section 2, the image reconstruction in EIT is briefly reviewed, and the wavelet-based fusion method for td- and fd-EIT images is demonstrated. In Section 3 and Section 4, both simulation and experimental results are presented to validate the proposed method with simulated or in vitro lung models. Finally, conclusions are drawn in Section 5.

## 2. Methodology

### 2.1. Principles of EIT

In this investigation the boundary was ∂Ω. Generally, for computational needs, it is assumed that Ω represents the body under the finite element model (FEM), Ω is discretized into small pixels or voxels and assigned a piecewise constant conductivity distribution, i.e., $\sigma ={\left[\sigma_{1},\ldots ,\sigma_{N}\right]}^{T}\in {\mathbb{R}}^{N}$ is assumed within Ω. The electric potential inside the sensing area induced by the injected AC current can be written as [5,6]:

(1) ∇·(σ(x,y)∇u(x,y))=0, (x,y)∈Ω

where *u* is the electric potential inside Ω. EIT is a nonlinear problem as the excitation current is a function of unknown conductivity distribution [21,22]. Therefore, based on the nonlinear model, the relation between the conductivity inside Ω and the boundary voltages at the boundary, ∂Ω, can be expressed as:

(2) V=U(σ)+e

where V is the measured voltage, U is the forward operator, and *e* is the additive noise and measurement error. Regarding the difference EIT imaging, Equation (2) can be linearized given a small conductivity perturbation Δσ, which can be expressed as (3):

(3) ΔV≈JΔσ

where J is the Jacobian matrix, and calculated by solving the EIT forward problem, i.e., Equation (1). Difference EIT calculates a vector of conductivity changes between the measured conductivity distribution and the reference conductivity distribution. One commonly employed method is the single frequency td-EIT imaging. More specifically, the AC current with selected frequency is injected into the sensing area. Hereafter, a measurement at time $t_{0}$ is acquired as the reference, and then the voltage change at time $t_{1}$ against the reference measurement is employed for image reconstruction, which is expressed as:

(4) $$\Delta V=V_{t_{1}}-V_{t_{0}}$$

In most cases, a set of reference measurements is acquired when the EIT sensor is filled with the background medium. To improve its precision, the measured data is typically averaged over many data frames.

Recently, incorporating the advances that have been made on the hardware system, a more attractive method in biomedical applications is the frequency-difference (fd-) EIT imaging. For the fd-EIT imaging, the current source is required to output a pair of complementary currents with two different frequency components, i.e., $f_{1}$ and $f_{2}$, which are injected to the sensor simultaneously or separately. At each frequency component, the boundary voltages are measured. The difference between the two sets of measured voltages is adopted for image reconstruction, which is calculated by

(5) $$\Delta V=V_{f_{2}}-V_{f_{1}}$$

After attaining the voltage difference, to solve the inverse or image reconstruction problem in EIT, a variety of algorithms have been proposed in the literature [23]. In general, using the regularization technique is the predominant way. The conductivity distribution can be obtained by solving the optimization problem:

(6) $$arg\;min\left\{\frac{1}{2}∥J\Delta \sigma -\Delta V{∥}_{2}^{2}+\lambda R\left(\Delta \sigma \right)\right\}$$

where R is the regularization function incorporating a priori knowledge and λ is the regularization parameter. In this case, for lung EIT, traditional and proprietary algorithms have been an obstacle to interpretation of EIT images as the reconstructed images cannot reveal the character of the lung [2]. GREIT (Graz consensus reconstruction algorithm for EIT) is a type of regularized imaging approach for 2D linear EIT reconstruction of the lung, which is based on the experience with a large number of linear regularized EIT reconstructions. One of the advantages of GREIT is that it conforms to the performance requirements for lung application, while other reconstruction algorithms do not. One can refer to Adler [2] for the detailed description of GREIT.

### 2.2. Wavelet-Based Image Fusion

**Figure 1:**
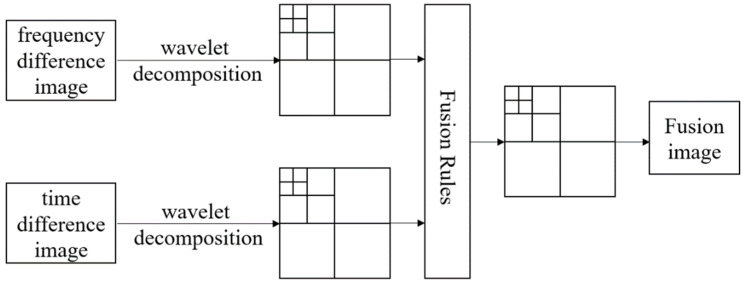
Image fusion process based on wavelet transform.

Image fusion has three levels: pixel level, feature level, and decision level [20]. Pixel-level fusion transcends other image fusion levels as it retains more detailed image information and can achieve higher accuracy. Among the pixel-level fusion methods, the wavelet-based method is recommended because of its non-redundancy and directionality [24,25,26,27]. Figure 1 shows the image fusion process based on the wavelet transform.

The fusion process is performed as follows: Firstly, the source images were decomposed by applying the wavelet transform with an appropriate wavelet basis function. In this procedure, reconstructed images by fd- and td-EIT can be assumed as a continuous change of two-dimensional signal $f\left(x_{1},x_{2}\right)\in L^{2}\left(R^{2}\right)$, $x_{1}$ and $\;x_{2}$ are its abscissa and ordinate, respectively. The wavelet transform of $f\left(x_{1},x_{2}\right)$ is expressed as:

(7) $$WT_{f}\left(a,b_{1},b_{2}\right)=\langle f\left(x_{1},x_{2}\right),\psi_{a,b_{1},b_{2}}\left(x_{1},x_{2}\right)\rangle$$

(8) $$\psi_{a,b_{1},b_{2}}\left(x_{1},x_{2}\right)=\frac{1}{a}\psi \left(\frac{x_{1}-b_{1}}{a},\frac{x_{2}-b_{2}}{a}\right)$$

where $\psi \left(x_{1},x_{2}\right)$ is a two-dimensional wavelet basis function, a is the scaling factor, and b is the displacement factor. By applying the basis function, the low-frequency and high-frequency components of the reconstructed image can be obtained, respectively. The low-frequency components of the image give approximate features, while the high-frequency components provide detailed features [27]. Secondly, appropriate fusion rules are employed to fuse the high frequency and low frequency components. The choices of fusion rules are critical in this procedure. There are many fusion rules, such as larger absolute value of coefficients, weighted average method, and local variance criterion [23]. According to previous studies, it is suggested that the fusion rules should adapt to the features of the source images. In this study, the fused image needs to maintain the position, size, edge, and shape of the target inclusions in the reconstructed images by fd- and td-EIT. In view of these requirements, the high frequency components were fused by taking the maximum absolute value in the source images. By combining the pixels correlation and regional variance in the image area, a hybrid fusion method was adopted for the fusion of low frequency components, as introduced by Wang et al. [27].

In the hybrid fusion rule, assuming that L(X) represents the low frequency coefficient matrix of the image  X , let G(X, p)  denote the regional variance of the region Q centred at p(m, n) in the low-frequency coefficient matrix of image X, which can be expressed as [27]:

(9) $$G\left(X,p\right)=\underset{q\subset Q}{\sum }w\left(q\right){\left|L\left(X,p\right)-\bar{u}\left(X,p\right)\right|}^{2}$$

where L(X,p) represents the value of the element at (m, n) in the low-frequency component coefficient matrix, and $\bar{u}\left(X,p\right)$ represents the average value of the elements inside Q. w(q) is the weight coefficient, which would increase if it approached p(m, n). $M_{2}\left(p\right)$ is the matching degree of the regional variance regarding the low-frequency coefficient matrix of the source images A and B at p(m, n), which is defined as:

(10) $$M_{2}\left(P\right)=\frac{2\sum_{q\subset Q}w\left(q\right)\left|L\left(A,p\right)-\bar{u}\left(A,p\right)\right|\left|L\left(B,p\right)-\bar{u}\left(B,p\right)\right|}{G\left(A,p\right)+G\left(B,p\right)}$$

It is noted that the value of $M_{2}\left(p\right)$ varies from 0 to 1. The smaller the $\;M_{2}\left(p\right)$, the lower the correlation between the low frequency coefficient matrices. Let $t_{2}$($0.5<t_{2}<1$) be the threshold of the matching degree. If $M_{2}\left(p\right)<t_{2}$, the option fusion strategy is adopted:

(11) $$L\left(F,p\right)=\left\{\begin{array}{c} L\left(A,p\right),\;\;\;G\left(A,p\right)\geq G\left(B,p\right)\\ L\left(B,p\right),\;\;\;G\left(A,p\right)<G\left(B,p\right) \end{array}\right\}$$

Otherwise, the average fusion strategy is used:

(12) $$L\left(F,p\right)=\left\{\begin{array}{c} W_{max}L\left(A,p\right)+W_{min}L\left(B,p\right),\;\;\;G\left(A,p\right)\geq G\left(B,p\right)\\ W_{min}L\left(A,p\right)+W_{max}L\left(B,p\right),\;\;\;G\left(A,p\right)<G\left(B,p\right) \end{array}\right\}$$

(13) $$W_{min}=0.5-0.5\left(\frac{1-M_{2}\left(p\right)}{1-t_{2}}\right)$$

(14) $$W_{max}=1-W_{min}$$

### 2.3. Image Quality Evaluation

Quantitative measures, e.g., relative coverage ratio (*RCR*), relative image error (*RIE*), and structural similarity (*SSIM*) index are defined to evaluate the quality of reconstructed images, which are the differences and correlations between the true image and the reconstructed image [28]:

(15) $$RCR=\frac{CR}{CR_{True}}$$

(16) $$RIE=\frac{∥\hat{g}-g∥}{∥g∥}$$

(17) $$SSIM=\frac{2\mu_{x}\mu_{y}\cdot 2\sigma_{x}\sigma_{y}}{\left(\mu_{x}^{2}+\mu_{y}^{2}\right)\left(\mu_{x}^{2}+\mu_{y}^{2}\right)}$$

where CR denotes the coverage ratio defined as the ratio of the size of the inclusions to the total size of the sensing area. Correspondingly, $CR_{True}$ is CR of the true target distribution. g and $\hat{g}$ are the true and reconstructed images, respectively, $\mu_{x}$ and $\mu_{y}$ are the mean grey values in the true and reconstructed images, respectively, with $\;\sigma_{x}$ and $\sigma_{y}$ being the corresponding standard deviations, respectively. Quantitative evaluation of the reconstructed images of biological objects under different frequencies is challenging because the conductivity changes with frequencies and the ground truth is unknown, especially for the fd-EIT imaging where different images can be obtained with different frequency intervals. Therefore, the popular criteria, such as image errors and correlation coefficients, cannot be employed in this case.

## 3. Characterization of td- and fd-EIT Imaging by Experiment

A series of static experiments were conducted to characterize the td- and fd-EIT imaging and validate the feasibility of the proposed method.

### 3.1. Experimental Setup

**Figure 2:**
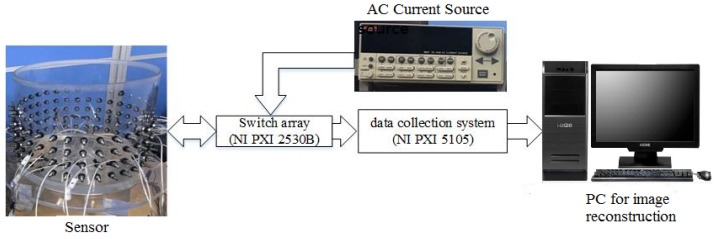
Experimental set-up.

An experimental system with a cylindrical tank sensor was established to characterize the fd- and td-EIT imaging, as shown in Figure 2. The diameter of the tank was 38 cm with four layers of EIT electrodes mounted axially. In each layer, 32 metallic electrodes (stainless steel screws, radius = 5 mm) were uniformly mounted on the inner surface of the tank. Sixteen of them (choosing one in the other two electrodes) in the second layer were employed for EIT imaging. A National Instruments (NI) PXI-based EIT system developed by Beihang University was employed in the experiment. The system mainly comprises three modules: the AC current source, the data collection system, and the PC with system control functions for image reconstruction. In the experiment, the cylindrical tank was filled with tap water as the background medium with a conductivity of 0.034 S/m. The pairwise injected current had a peak-to-peak magnitude of around 1 mA, while the frequency was set to 1 KHz, 5 KHz, 10 kHz, 20 kHz, and 50 KHz. With the adjacent excitation strategy, the excitation current was injected into a pair of adjacent electrodes. By multiplexing, the potential differences between each possible pair of adjacent electrodes were measured by the data collection system. The measurements were then sent to the PC for image reconstruction via a USB interface. Finally, an image of the target inclusions was reconstructed in MATLAB using the data received. For a single measurement, 20 frames of data were averaged to mitigate the measurement noise.

**Figure 3:**
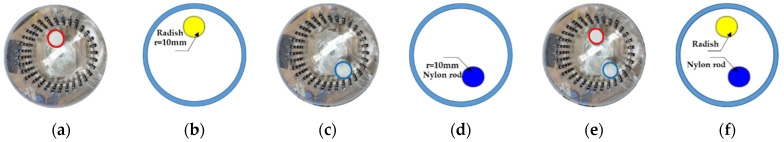
Experimental setup of three different phantoms. (**a**) Phantom 1, peeled radish; (**b**) distribution of phantom 1; (**c**) phantom 2, nylon rod; (**d**) distribution of phantom 2; (**e**) phantom 3, peeled radish and nylon rod; and (**f**) distribution of phantom 3.

Td- and fd-EIT were used to image three different phantoms, which were set up by inserting a conductive inclusion (a peeled radish in phantom 1), a non-conductive inclusion (a nylon rod in phantom 2), and both of them (in phantom 3) at a certain location in the tank. The diameters of the peeled radish and nylon rod were 60 mm and 55 mm, respectively. Their length was sufficiently long to extend beyond the water surface so that all tests were conducted with target inclusions homogeneous in the vertical direction. Figure 3a–f show the phantom pictures and corresponding distributions of the inclusions, respectively.

**Figure 4:**
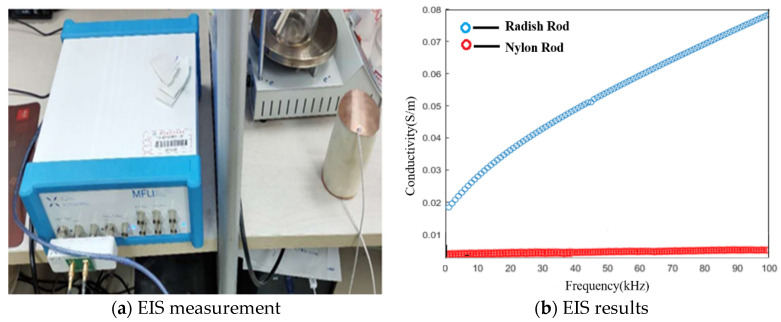
Electrical impedance spectroscopy of peeled radish and nylon.

According to previous work [29,30], electrical impedance spectroscopy (EIS) can reveal the frequency response of electrical impedance of bio-tissues, which can be employed to demonstrate the frequency-dependencies of the conductivities of the peeled radish and nylon rod. Figure 4 shows the EIS measurements of the peeled radish and nylon rod by an impedance analyzer (MFLI Lock-in Amplifier-500 kHz/5 MHz, Zurich Instruments, Zurich, Switzerland) over the selected frequency range, i.e., 0–100 kHz. The results of EIS indicated that the conductivity of the radish increased linearly with the increase in frequency, and the linear fitting formula was:

(18) σ=0.000563×f+0.02374

Its RMSE (root mean squared error) was 0.001834, and the value of R-square (coefficient of determination) was 0.9877.

In particular, it is noted that the conductivity of the radish at 10 kHz was about 0.03 S/m, which is nearly the same as the conductivity of the background medium. With respect to the nylon rod, the EIS data illustrate that its conductivity stayed almost unchanged with frequency. This is consistent with the previous analysis and verifies the feasibility of the use of the radish and nylon rod for the dedicated study on td- and fd-EIT imaging.

### 3.2. Experimental Results and Analysis

**Figure 5:**
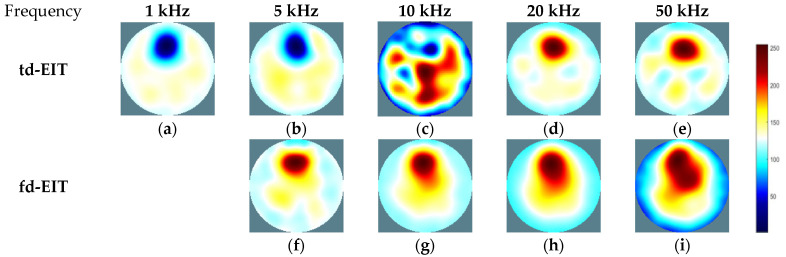
Td- and fd-EIT imaging results of phantom 1. (**a**–**e**) are td-EIT images of phantom 1 at 1, 5, 10, 20 and 50 kHz, respectively; (**f**–**i**) are fd-EIT images of phantom 1 at 5, 10, 20 and 50 kHz, respectively.

**Figure 6:**
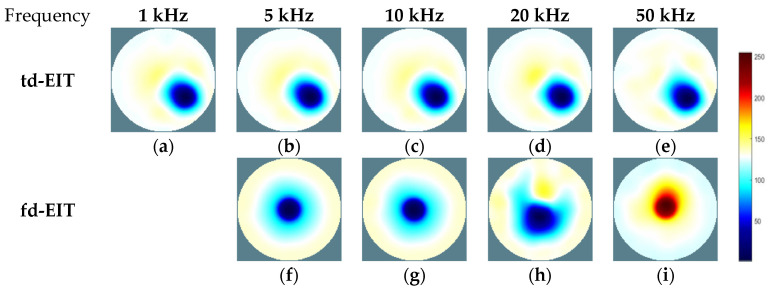
Td- and fd-EIT imaging results of phantom 2. (**a**–**e**) are td-EIT images of phantom 2 at 1, 5, 10, 20 and 50 kHz, respectively; (**f**–**i**) are fd-EIT images of phantom 2 at 5, 10, 20 and 50 kHz, respectively.

**Figure 7:**
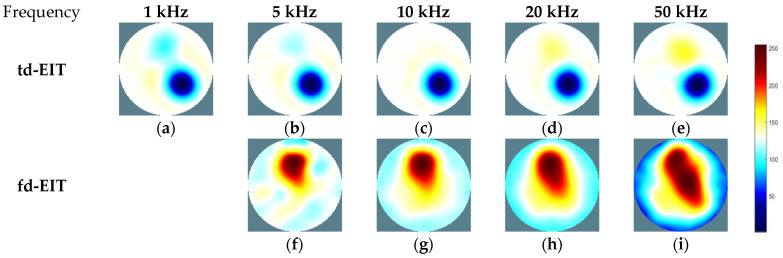
Td- and fd-EIT imaging results of phantom 3. (**a**–**e**) are td-EIT images of phantom 3 at 1, 5, 10, 20 and 50 kHz, respectively; (**f**–**i**) are fd-EIT images of phantom 3 at 5, 10, 20 and 50 kHz, respectively.

By applying the GREIT algorithms for image reconstruction, the td- and fd-EIT imaging results of the three phantoms were attained, and the size of the images w 64×64 pixels. In fd-EIT, 1 kHz was selected as the reference frequency. The voltage differences between the measured voltages at all others frequencies and at the reference frequency were procured for image reconstruction. In td-EIT, the measured voltage when the EIT sensor was filled with the background medium was taken as the reference, i.e., the measurement at $t_{1}$, and the voltage differences were obtained when perturbations were present in the background medium under the tested frequencies. The td- and fd-EIT imaging results are shown in Figure 5, Figure 6 and Figure 7.

**Figure 8:**
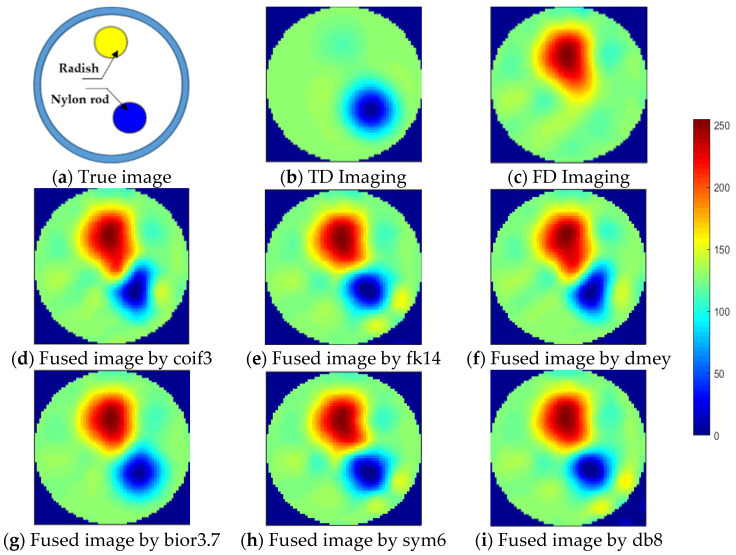
Fused images by applying the wavelet-based fusion method: (**a**) Ture image, (**b**) TD imaging, (**c**) FD imaging, (**d**) fused image by coif3, (**e**) fused image by fk14, (**f**) fused image by dmey, (**g**) fused image by bior3.7, (**h**) fused image by sym6, and (**i**) fused image by db8.

Figure 5 and Figure 6 show the td- and fd- imaging results of phantoms 1 and 2 at 1 kHz, 5 kHz, 10 kHz, 20 kHz, and 50 kHz. Figure 5, Figure 6 and Figure 7 have the same color bars, and the gray value range was 0–255, corresponding to the conductivity value of 0–0.08 S/m, respectively. The same applied to the relationship between the gray value and the conductivity in Figure 8, despite of the use of different colors for presentation. As indicated, td-EIT can reconstruct the position and shape of the single radish or nylon rob clearly (except for at 10 kHz where the conductivity of radish was close to the background medium as indicated by td-EIT imaging results). Fd-EIT could image the radish in phantom 1, but it failed to indicate the accurate position of the nylon rod in the reconstructed image since the conductivity of non-conductive nylon rod was not frequency-dependent. Regarding the phantoms 1 and 2, td-EIT provided better imaging results of the inclusions, as indicated in Table 1 and Table 2 for quantitative evaluation, where td-EIT imaging results have lower RIE, as well as RCR and SSIM closer to 1. In Figure 5, td-EIT imaging results indicate that the color scale of the reconstructed inclusion changed with the increase in excitation frequency, which was due to the frequency-dependency of the conductivity of peeled radish. More specifically, the conductivity of peeled radish was lower than the tap water when the frequency was lower than 10 kHz, and larger than the tap water at frequencies >10 kHz. On the other hand, since the conductivity of the radish was related to frequency, the image quality was sensitive to the frequency intervals. Besides, size overestimation and image distortion are more significant with increase in the frequency intervals, which is attributed to the decrease of SNR with the increase in frequency. The image distortion was the most serious at 10 kHz due to the conductivity of peeled radish being very close to the tap water and the measured voltages had a much smaller signal-to-noise ratio (SNR), which would lead to wrong imaging result since the EIT reconstruction was ill-conditioned.

For further demonstration, imaging results of phantom 3 by td- and fd-EIT are shown Figure 7. In Figure 7, the position and shape of the nylon rod can be well reconstructed by td-EIT, while due to the existence of the non-conductive nylon rod, the radish can hardly be seen as it causes much smaller responses at the sensor boundary. However, regarding fd-EIT imaging, only the peeled radish is shown in the reconstructed images. The reason is that the conductivity of the radish varies with excitation frequency while that of the nylon rod does not. These findings are verified by the evaluation metrics in Table 3.

### 3.3. Image Fusion with Respect to td- and fd-EIT Imaging

To evaluate the performance of the above-mentioned fusion strategy, the reconstructed images of phantom 3 by td- and fd-EIT at 5 kHz were taken as the source images. Meanwhile, to investigate the impact of wavelet basis function, various basis functions available in MATLAB were employed in the wavelet transform, including “coif3”, “fk14”, “bior3.7”, “dmey”, “sym6”, and “db8”.

As shown in Figure 8, the source images by td- and fd-EIT cannot either demonstrate the conductive inclusion (i.e., the radish) or the non-conductive inclusion (i.e., the nylon rod). By applying the wavelet-based image fusion strategy, the fused image can successfully recover both the conductive and non-conductive inclusions, which resolves the issues of td- and fd-EIT imaging. It is noted that different basis functions have significant impact on the quality of the fused images, especially for the radish. By employing the “coif3” or “dmey” basis function in the wavelet transform, the shapes of the nylon rob in the fused images are obviously distorted besides the shapes of the radish. Overall, the “fk14”, “bior3.7”, “sym6”, and “db8” basis functions can provide fused images of reasonable quality, which are much better than those reconstructed by either td-EIT or fd-EIT.

## 4. Investigation for Improving Lung Ventilation Imaging with Thorax Model

**Figure 9:**
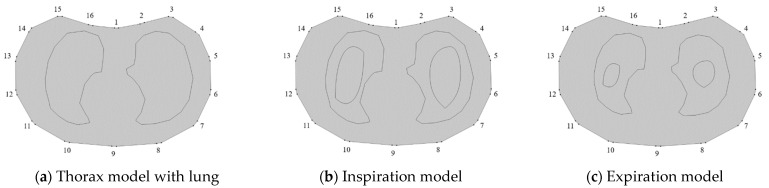
2-D view of thorax model built in simulation. (**a**) Thorax model with lung, (**b**) inspiration model, and (**c**) expiration model.

To investigate the performance of the proposed method in medical EIT imaging of lung ventilation, simulations and in vitro experiments were conducted. A real-shaped thorax model, as shown in Figure 9, was built for FEM simulation. The thorax domain and the sub-domains of the lungs were created according to the build-in function of the thorax model in EIDORS [31]. It is well known that the whole respiratory cycle contains two stages: inspiration and expiration. In the simulation, the lung was simulated as varying states by setting different air contents in the given 2-D lung model. The model is assumed ideal as it does not consider the modelling errors regarding the non-homogenous background, the lung shape movement and the change of the chest due to the breathing in the real clinical situations. The following two test cases were designed:

Case 1: Lung imaging in the inspiration (Figure 9b) stage, during which the breathing would increase the air content, and air would almost fully fill the lung in the end of inspiration. For this reason, it is suggested to set up a sufficiently large amount of air content inside the lung model to simulate the inspiration.

Case 2: Lung imaging in the expiration (Figure 9c) stage, during which the air content decreases with the breathing and becomes negligible in the end of expiration. In a similar fashion, a small amount of air content was set up inside the lung model to simulate the expiration.

In the simulation, lung conductivity was considered as a function of the excitation frequency, according to literature [32,33]. The maximum and minimum conductivity values were set to 0.079 S/m and 0.11 S/m, respectively. The conductivity of background tissues was set to 0.2 S/m, as suggested by Liu et al. [6]. Sixteen electrodes were attached on the boundary of the thorax domain. The same current injection and measurement strategy employed in the previous experimental studies was also adopted in the simulation. The simulation was conducted with 2-D FEM approximation in COMSOL. To produce a reference for fd- and td-EIT imaging, the reference frequency was chosen to be 1 kHz, and the reference distribution for td-EIT imaging was assumed to be the thorax model only containing the background medium and lung tissues, as shown in Figure 9a.

**Figure 10:**
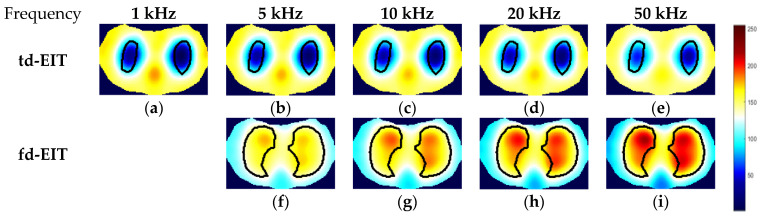
Td- and fd-EIT imaging results in case 1 with simulated lung. (**a**–**e**) are td-EIT images in case 1 at 1, 5, 10, 20 and 50 kHz, respectively; (**f**–**i**) are fd-EIT images in case 1 at 5, 10, 20 and 50 kHz, respectively.

**Figure 11:**
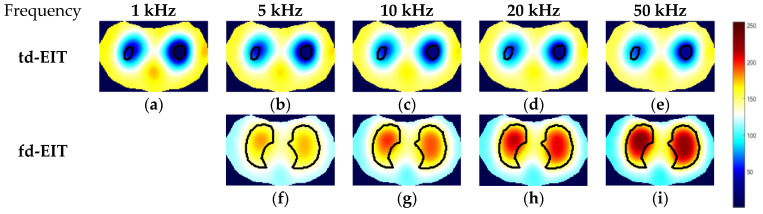
Td- and fd-EIT imaging results in case 2 with simulated lung. (**a**–**e**) are td-EIT images in case 2 at 1, 5, 10, 20 and 50 kHz, respectively; (**f**–**i**) are fd-EIT images in case 2 at 5, 10, 20 and 50 kHz, respectively.

Simulation results are shown in Figure 10 and Figure 11. In the reconstructed images, whose size is 84×128 pixels, the outermost dark solid line and the innermost solid dark line denote the true lung shape and air region, respectively. In the simulated cases 1 and 2, the fd- and td-EIT imaging can successfully reconstruct the lung-shaped inclusion or the air inclusion, respectively, but not both of them. In addition, the air inclusion is overestimated by td-EIT, while the lung-shaped inclusion is underestimated by fd-EIT. In fact, the end of expiration would reduce the content of air in the lung, and hence result in a higher RCR (the air domain is significantly overestimated) in the case 2, as shown in Table 4 and Table 5. Due to the limitation of the reconstruction algorithm, the sharp edge and accurate shape of the inclusions were not well preserved in the reconstructed images. Note that the fd-EIT images at 1 kHz cannot be obtained since it is the chosen reference frequency.

**Figure 12:**
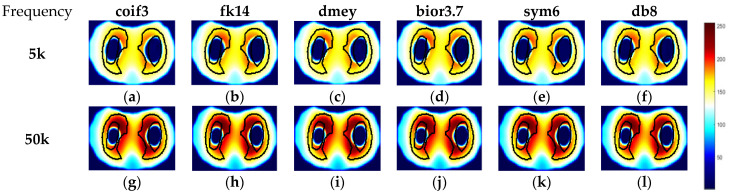
Fused images by applying wavelet-based fusion method in case 1. (**a**–**f**) are fused images using different wavelet bases in case 1 at 5 kHz, respectively; (**g**–**l**) are fused images using different wavelet bases in case 1 at 50 kHz, respectively.

**Figure 13:**
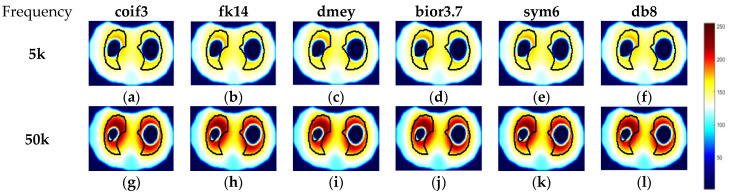
Fused images by applying wavelet-based fusion method in case 2. (**a**–**f**) are fused images using different wavelet bases in case 2 at 5 kHz, respectively; (**g**–**l**) are fused images using different wavelet bases in case 2 at 50 kHz, respectively.

The reconstructed images by td- and fd-EIT at 5 kHz and 50 kHz were used to demonstrate the improvement of the lung imaging by the proposed fusion method. As shown in Figure 12 and Figure 13, the image fusion for lung imaging was fairly successful. Compared with the imaging results by either td-EIT or fd-EIT, the fused images cannot only provide the rational changes of air content, but also the position and shape of the lung-shaped inclusion. It is not trivial in practice to obtain the change of air content and lung shape information simultaneously since the latter can serve as a reference and there may, in addition to the air current, be tissue changes related to certain diseases such as pulmonary edema. Meanwhile, the size and shape of the air inclusion or lung-shaped inclusion in the fused image agree well with the actual ones. It is noted that the fused images at 50 kHz indicate a much larger image contrast in terms of the lung shape. This is in accordance with the finding that the conductivity variation of the lung tissues between the measurement frequency (5 kHz and 50 kHz) and the reference frequency (1 kHz) increases with frequency.

**Figure 14:**
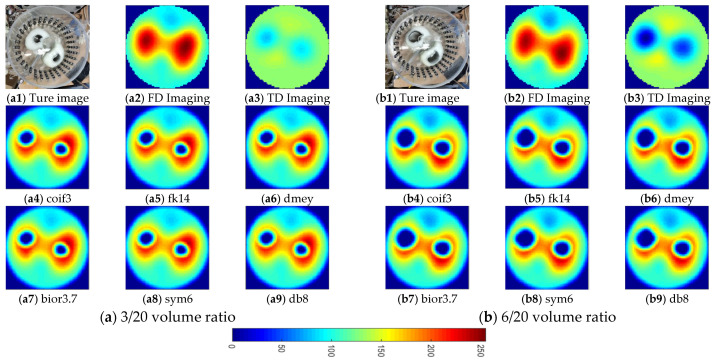
Reconstructed and fused images of lung-shaped white gourd containing a varied content of air at 10 kHz. (**a**) 3/20 volume ratio: (**a1**) is the actual experimental image; (**a2**,**a3**) are fd-EIT and td-EIT images, respectively; (**a4**–**a9**) are fused images using different wavelet bases, respectively. (**b**) 6/20 volume ratio: (**b1**) is the actual experimental image; (**b2**,**b3**) are fd-EIT and td-EIT images, respectively; (**b4**–**b9**) are fused images using different wavelet bases, respectively.

**Figure 15:**
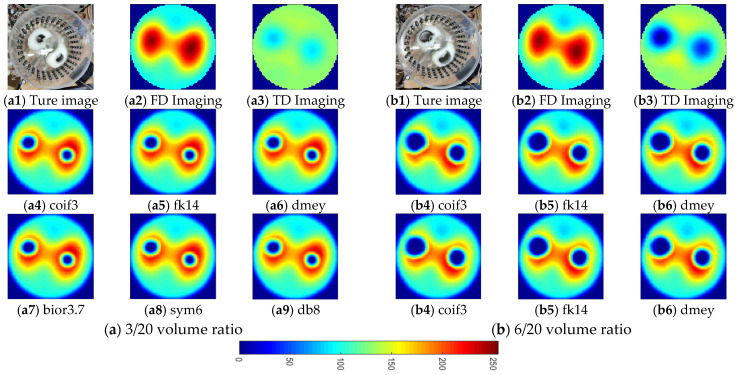
Reconstructed and fused images of lung-shaped white gourd containing a varied content of air at 20 kHz. (**a**) 3/20 volume ratio: (**a1**) is the actual experimental image; (**a2**,**a3**) are fd-EIT and td-EIT images, respectively; (**a4**–**a9**) are fused images using different wavelet bases, respectively. (**b**) 6/20 volume ratio: (**b1**) is the actual experimental image; (**b2**,**b3**) are fd-EIT and td-EIT images, respectively; (**b4**–**b9**) are fused images using different wavelet bases, respectively.

To further validate the feasibility and effectiveness of the proposed fusion method, static in vitro experiments were conducted. These experiment was conducted in the same tank as in the previous experiments. The tank was also filled with tap water as the background medium, and two lung-shaped inclusions were made of white gourd. Two pairs of air-containing inclusions were inserted into the two lung-shaped inclusions to simulate the lung-filling conditions in the expiration and inspiration stages, respectively, as in the two simulation cases. Figure 14 and Figure 15(a1,b1) show the phantom pictures. To quantify the air content inside the lung-shaped inclusion, the volume ratio was measured. The volume ratio was 3/20 and 6/20 for the expiration and inspiration setup, respectively.

Based on the visual inspection of Figure 14 and Figure 15, the fused images provide a reasonable estimation of the shape and location of the lung-shaped inclusion as well as the air inclusion inside it at different frequencies. The td-EIT performs well in reconstructing the air inclusion, while the fd-EIT is better in imaging the lung-shaped inclusion. It is noted that the contrast variation caused by the change of air content is clearly shown. Therefore, it can be concluded that the lung imaging based on the proposed fusion method is feasible and effective.

## 5. Conclusions

This paper characterizes and compares fd- and td-EIT for the imaging of target distributions composed of bio-material inclusions and non-conductive inclusions, and the lung ventilation imaging is a potentially applicable scenario. Through an experimental study with a peeled radish or a nylon rod as targets, it was found that td-EIT reconstructions can reconstruct the bio-material inclusion (the radish) and the non-conductive inclusion (the nylon rod) if either the former or the latter exists in the sensing domain. Fd-EIT could only image the bio-materials inclusion (the radish) as the conductivity of non-conductive materials is not frequency-dependent, but has significant distortions with an appropriate frequency interval. However, both the td-EIT and fd-EIT failed to simultaneously reconstruct the radish and nylon rod if both of them exist in the sensing domain. This is because the response to the non-conductive rod (i.e., a much larger conductivity contrast to the background medium) would overwhelm that to the radish for td-EIT imaging, which would occur when imaging certain lung diseases such as pulmonary edema. To tackle with this dilemma, it is proposed to fuse the td- and fd-EIT imaging results using a dedicated wavelet-based fusion strategy, which can integrate the merits of td- and fd-EIT. Based on the simulation and experiment with lung-shaped inclusions or in vitro lung models, it was found that the proposed method has much better reconstruction accuracy than either td- or fd-EIT, which can simultaneously reconstruct both the lung-shaped inclusions and air inclusions in the fused image. Meanwhile, the proposed method can reduce the image distortions by td- or fd-EIT, and provide the relative shapes, sizes, and positions of those inclusions. In summary, the proposed method can improve the performance of EIT in lung ventilation imaging, so that the diagnosis functions of EIT can be expanded to deal with more lung-tissue-related diseases. Although such a fd- and td-EIT system certainly requires the hardware to accommodate to both the EIT modes, which can be readily achieved in practical applications, this paper aims to determine how much improvement can be expected by applying such a fusion method and understanding and identifying the compromises involved in fd- and td-EIT imaging. Future work will focus on optimizing the image reconstruction and fusion methods to further enhance the image quality. On other hand, the proposed method should be tested in more realistic cases of lung imaging.

## Figures and Tables

**Table 1:** RCR, RIE, and SSIM regarding td- and fd-EIT imaging results of phantom 1.

Frequency		1 kHz	5 kHz	10 kHz	20 kHz	50 kHz
**RCR**	**fd-EIT**	\	0.8309	1.1268	1.8621	4.0221
	**td-EIT**	0.7463	0.6415	\	1.1047	1.0386
**RIE**	**fd-EIT**	\	0.3933	0.4017	0.2950	0.4088
	**td-EIT**	0.0893	0.1156	\	0.2160	0.2496
**SSIM**	**fd-EIT**	\	0.7648	0.6764	0.4385	0.1614
	**td-EIT**	0.8039	0.7071	\	0.8583	0.6943

**Table 2:** RCR, RIE, and SSIM regarding td- and fd-EIT imaging results of phantom 2.

Frequency		1 kHz	5 kHz	10 kHz	20 kHz	50 kHz
**RCR**	**fd-EIT**	\	1.1667	1.1667	1.4118	1.0588
	**td-EIT**	1.0172	1.0221	1.0147	0.9706	1.0049
**RIE**	**fd-EIT**	\	0.1407	0.1421	0.1850	0.4487
	**td-EIT**	0.1017	0.0961	0.0964	0.1157	0.0882
**SSIM**	**fd-EIT**	\	-0.0272	−0.0272	−0.0284	−0.0262
	**td-EIT**	0.7544	0.7549	0.7578	0.7574	0.8169

**Table 3:** RCR, RIE, and SSIM regarding td- and fd-EIT imaging results of phantom 3.

Frequency		1 kHz	5 kHz	10 kHz	20 kHz	50 kHz
**RCR**	**fd-EIT**	\	1.0129	1.3462	2.1952	4.7882
	**td-EIT**	1.1007	1.1032	1.1106	1.1253	1.1327
**RIE**	**fd-EIT**	\	0.3862	0.4052	0.3079	0.4346
	**td-EIT**	0.0986	0.0869	0.0844	0.1096	0.1330
**SSIM**	**fd-EIT**	\	0.7832	0.7071	0.4318	0.1339
	**td-EIT**	0.8939	0.8998	0.8912	0.8930	0.8930

**Table 4:** RCR and RIE regarding td- and fd-EIT imaging results in case 1.

Frequency		1 kHz	5 kHz	10 kHz	20 kHz	50 kHz
**RCR**	**fd-lung**	\	0.9063	0.9137	0.9207	0.9470
	**td-air**	1.2380	1.1077	1.0575	1.0010	0.9381
**RIE**	**fd-EIT**	\	0.4030	0.4032	0.4034	0.4041
	**td-EIT**	0.2395	0.2354	0.2339	0.2325	0.2308

**Table 5:** RCR and RIE regarding td- and fd-EIT imaging results in case 2.

Frequency		1 kHz	5 kHz	10 kHz	20 kHz	50 kHz
**RCR**	**fd-lung**	\	0.9064	0.9145	0.9237	0.9446
	**td-air**	4.1290	3.3334	2.9910	2.6587	2.2256
**RIE**	**fd-EIT**	\	0.4169	0.4168	0.4169	0.4172
	**td-EIT**	0.2232	0.2214	0.2208	0.2204	0.2200

## Data Availability

Not applicable.
